# Study on the failure mechanism and improvement measures of bearings in the full-ocean-depth ultra-high-pressure seawater pump

**DOI:** 10.1038/s41598-025-25518-y

**Published:** 2025-11-24

**Authors:** Zhida Chen, Xiguang Hu, Linglong Li, Cong Ye, Defa Wu, Shuai Liu, Yunxiang Ma, Lang Gu

**Affiliations:** 1 State Key Laboratory of Deep-sea Manned Vehicles, Ship Scientific Research Center, Wuxi, 214082 China; 2 State Key Laboratory of Intelligent Manufacturing Equipment and Technology, Huazhong University of Science and Technology, Wuhan, 430074 China

**Keywords:** Engineering, Materials science

## Abstract

Under ultra-high-pressure full-ocean-depth conditions, the rolling bearings of seawater pumps are often subjected to coupled stress conditions, including high external pressure, oil–water emulsification, and sustained high loads. Early failure tends to occur, which severely compromises system stability and reliability. This study focuses on identifying the typical failure mechanisms of bearings and proposing key optimization measures. A high-pressure experimental system rated at 120 MPa was constructed. Long-term water injection and drainage cycling tests were performed, followed by teardown inspections of failed prototypes. The bearing degradation was found to involve multiple failure modes, including rolling element fracture, cage breakage, lubricant emulsification, and three-dimensional embedded abrasive wear. The combined effects of lubricant degradation and particulate contamination primarily caused these failures. Comparative tests were conducted on ceramic bearings, PEEK bearings, and tapered roller bearings. The results confirmed that the tapered roller bearing exhibited superior environmental adaptability under lubrication with No. 10 aviation hydraulic oil. To enhance system performance, two engineering measures were proposed: (1) the use of heavy-duty tapered roller bearings to increase load capacity and fatigue life; (2) the addition of molybdenum disulfide (MoS₂) anti-wear additives to the lubricant to improve lubrication stability and wear resistance. Validation results showed that, after optimization, the prototype achieved significantly higher mechanical efficiency under 120 MPa conditions, and bearing wear was substantially reduced. These findings provide theoretical support and engineering guidance for selecting bearings and developing lubrication strategies in high-pressure, deep-sea hydraulic systems.

## Introduction

With the increasing demands of deep-sea operations for long-duration, high-reliability, and environmentally friendly lubrication, the rolling bearing in seawater pumps—core power components of deep-sea hydraulic systems—has become a critical limiting factor for overall system reliability. Under full-ocean-depth conditions, the bearing is not only exposed to prolonged ultra-high hydrostatic pressure but also subjected to harsh coupled effects such as reciprocating loads, lubrication degradation, and particle erosion, leading to a high risk of early failure. In particular, in water-based or pure-water hydraulic systems, emulsification and viscosity reduction of the lubricant accelerate film breakdown and contact fatigue, directly affecting service life and maintenance intervals.

The lubrication characteristics and failure behaviors of bearings have long been focal points in both academic and engineering fields. Prior studies have achieved notable progress in oil film mechanics, thermo-frictional effects, and fatigue life modeling. For example, Tian, et al^1^. established stiffness and damping models for thrust bearing oil films based on hydrodynamic lubrication theory. Kamarapu, et al^2^. investigated the thermal conductivity of bio-based nano-lubricants and demonstrated effective reduction of roller bearing wear under fatigue loads. Wu, et al^3^. proposed a dynamic model for ceramic bearings with elastohydrodynamic lubrication (EHL) effects, highlighting the influence of temperature fluctuations on film pressure and thickness. Mo, et al^4^. further integrated multi-physics coupling among thermal, lubrication, and dynamic fields to enhance model accuracy.

Researchers have investigated failure modes under corrosion, contamination, and complex loading. Lu, et al^5^. revealed damage patterns of propeller hub bearings under service conditions. Zhang, et al^6^. proposed a nonlinear framework to evaluate post-buckling and dynamic responses of piezoelectric laminated shells. Li, et al^7^. developed a spline-based TMS method considering fretting friction for spline–shaft systems. Yang, et al^8^. modeled resonance behaviors of bearing–rotor systems with flexible supports. Li, et al^9^. constructed a double-row cylindrical roller bearing model with irregular defects to study the influence of defect size and loading on system response.

In the domain of intelligent monitoring and fault diagnosis, a variety of AI-assisted signal processing techniques have emerged. These include: self-powered triboelectric lubrication monitoring systems^10^, novel oil supply structures for high power density bearings^11^, life prediction models for solid-lubricated bearings^12^, wear modeling under oil–gas lubrication^13^, and composite bearing design methodologies^14^. Hu, et al^15^. established a TEHD-based coupled simulation model with optimized temperature measurement strategies. Hong, et al^16^. correlated surface morphology and finite element analysis to identify structural failure causes. Tian, et al^17^. developed a nonlinear model for cage fracture to characterize fault evolution. Salunkhe, et al. proposed a series of bearing fault diagnosis methods, which greatly promoted the progress of bearing fault diagnosis research^18–23^.

In terms of life prediction, significant advances have also been made. Kong and Li^24^and Wang, et al^25^. proposed remaining useful life (RUL) estimation methods based on limited samples. Dai, et al^26^. combined wavelet kernel networks with bidirectional gated recurrent units (Bi-GRU) to build a robust life evaluation system. Fault diagnosis strategies have been extended by Schmidt, et al^27^. for speed-varying conditions. Shinde and Desavale^28^applied MMDA and SVM for rotor–bearing system imbalance and misalignment detection. Vishwendra, et al^29^. proposed vibration feature classification for deep groove ball bearing defects. Yang, et al^30^. combined crack propagation path simulation and fracture surface validation to investigate failures in intershaft bearings of turbofan engines. Shinde, et al^31^. used the K-NN algorithm for intelligent bearing fault classification. Shi, et al^32^. introduced stiffness-equivalent modeling to improve the diagnosis of cylindrical roller bearings. Shinde, et al^33^. further explored the synergy between FEM modeling and MMDA for contact stress identification. Building upon these foundations, recent studies have expanded the understanding of bearing degradation by addressing contact fatigue^34^, diagnostic strategies^35–37^, Hertzian contact stress distributions^38^, and noise interference analysis^39^.

Despite these advances in modeling, monitoring, and prediction, a systematic analysis of degradation mechanisms under complex oil–water environments remains limited. In particular, the effects of lubricant emulsification and viscosity decay on surface fatigue and crack propagation under high-pressure conditions in the deep sea have not been quantitatively studied. The role of structural optimization in altering lubrication boundary conditions also remains unclear, which hinders the direct application of current results to ultra-high-pressure seawater pumps in full-ocean-depth environments.

To bridge this gap, this study investigates tapered roller bearings used in ultra-high-pressure seawater pumps. Through long-term operation and post-test disassembly, typical failure modes were identified, including severe oil emulsification, contact fatigue cracks, rolling element fragmentation, cage breakage, and embedded particle wear. Based on the observed phenomena, a degradation path framework was developed considering multi-physics coupling under deep-sea conditions. In response to the identified issues, two engineering strategies are proposed: (1) adding molybdenum disulfide (MoS₂) anti-wear additives to No. 10 aviation hydraulic oil to enhance film load capacity and thermal stability; and (2) using heavy-duty tapered roller bearings to improve fatigue life and shock resistance. Experimental validation demonstrated that these measures significantly improved lubrication conditions under high pressure, increased mechanical efficiency, and effectively mitigated early failure. This research provides a more complete understanding of bearing degradation in oil–water systems and offers practical technical solutions for bearing selection and lubrication optimization in ultra-deep-sea hydraulic pumps.

## Analysis of bearing failure phenomena and mechanisms

### Target configuration and baseline setup

The studied system is a full-ocean-depth ultra-high-pressure seawater pump. The rated rotational speed is 1500 rpm. The pump operates inside a pressure chamber, undergoing external pressure cycling at 120 MPa. The key support components in the transmission chain are tapered roller bearings: SKF 30,206 (baseline configuration) and SKF 33,206 (improved configuration), with one set mounted on each of the front and rear end caps.

The maximum radial force generated by a single plunger is approximately 6 kN. After force distribution through the internal mechanism, the maximum radial load acting on a single bearing does not exceed 4.5 kN. The operating temperature conditions are characterized by a “cold-outside–hot-inside” gradient, with seawater temperatures ranging from 2 to 4 °C, while the internal cavity experiences a temperature rise during operation.

In terms of load-bearing and life constraints, the design priority is to increase the factor (C/P)^^*p*^, where *p* = 10/3 for tapered roller bearings. Under the same radial load P, the basic dynamic load rating C increases from 50 kN (SKF 30206) to 79.7 kN (SKF 33206). This corresponds to a theoretical bearing life increase by a factor of approximately:

1 $$\frac{{{L_{10}}(33206)}}{{{L_{10}}(30206)}}={\left( {\frac{{79.7}}{{50}}} \right)^{10/3}} \approx 4.73,$$

Contact and Lubrication Constraints: Under high external pressure and boundary lubrication risks, bearing types with longer rollers and larger line contact areas are preferred. This selection helps to reduce local contact stress and the PV value. The viscosity ratio κ should be maintained at ≥ 1, with a recommended range of 1 to 4, to ensure adequate lubrication performance.

Medium and Temperature Compatibility: At low ambient temperatures (2–4 °C), high viscosity can lead to poor lubricant flowability, while a temperature rise during operation may result in rapid viscosity loss. Therefore, the selected lubricant must possess both low-temperature fluidity and high-temperature viscosity retention. Additionally, strong anti-emulsification properties are required to resist the adverse effects of minor seawater intrusion.

#### Boundary description of rejected configurations

Ceramic Bearings: Ceramic bearings offer excellent corrosion resistance and low friction. However, under transient impact and axial load coupling conditions in seawater pump operations, the risk of brittle fracture is significant, and the consequences of fragmentation are unacceptable. Moreover, under low-viscosity water lubrication, it is difficult to maintain an adequate elastohydrodynamic lubrication (EHL) film. The risk of κ ≪ 1 is pronounced. In-field solid contaminants, such as sand or metallic particles, can further aggravate third-body abrasion.

PEEK Bearings: PEEK bearings exhibit good corrosion resistance and moderate thermal tolerance. However, their load-carrying capacity and dimensional stability are inferior to those of metallic roller bearings. Under prolonged high-load or high-speed conditions, deformation and wear are more likely to occur. In deep-sea, low-temperature water-lubricated environments, similar challenges exist, including skinny lubrication films and increased sensitivity to particle-induced wear.

#### Structural configuration and parameter settings of the adopted scheme

Bearing Type: Tapered roller bearings were adopted, featuring line contact, extended roller length, and combined axial-radial load capacity. The baseline model was SKF 30,206, and the upgraded version was SKF 33,206. The contact length was increased from 9.33 mm to 16.2 mm, which helped reduce local contact stress and the PV index. Lubrication film stability and fatigue resistance were thereby improved.

Load and Life Performance: Under radial load conditions not exceeding 4.5 kN, the (C/P)^^*p*^ ratio was significantly enhanced. Based on this improvement, a rated life extension of approximately 4.73 times was achieved, contributing to greater reliability under ultra-high-pressure conditions.

Relevance to Failure Analysis: The improved bearing configuration ensured sufficient structural and load-bearing redundancy. This allowed degradation of lubrication—such as emulsion formation and κ < 1 conditions—to be more clearly identified as the dominant failure cause. The possibility of confusion between “insufficient mechanical capacity” and “lubrication degradation” was thus effectively avoided.

The selected bearing for the initial configuration was the tapered roller bearing model 30,206, made of GCr15 bearing steel. This material is commonly used in high-load rolling elements due to its excellent mechanical strength and wear resistance. To support the subsequent failure analysis, the key mechanical properties of GCr15 are summarized as follows: Young’s modulus = 210 GPa, yield strength ≈ 2550 MPa, and surface hardness = 62–64 HRC. These properties make it suitable for withstanding high cyclic contact stress and surface fatigue loading, particularly in ultra-high-pressure seawater pump environments.

#### Lubricant type and performance indicators

In the original configuration, No. 10 aviation hydraulic oil was selected as the primary lubricant for the seawater pump bearing system. This choice was based on the lubricant’s superior physicochemical properties under extreme environmental conditions. Specifically, No. 10 aviation oil exhibits excellent low-temperature fluidity, which ensures effective lubrication at deep-sea ambient temperatures. Additionally, it demonstrates high oxidation resistance and outstanding anti-emulsification stability, allowing it to retain performance even when exposed to high-pressure seawater ingress.

Compared to conventional mineral-based lubricants, No. 10 aviation hydraulic oil maintains a more stable kinematic viscosity profile under varying pressure and temperature conditions. It is also more compatible with stainless-steel and bearing alloy materials typically used in deep-sea equipment, reducing the risk of chemical degradation or tribo-chemical reactions. These properties collectively contribute to prolonged bearing life, improved mechanical efficiency, and reduced risk of lubrication failure during full-ocean-depth operation.

No. 10 aviation hydraulic oil was selected as the working lubricant for the bearing system. This oil offers excellent low-temperature fluidity, high oxidative stability, and strong resistance to emulsification under high-pressure seawater exposure, making it well-suited for deep-sea applications. Compared with conventional mineral-based lubricants, it maintains better kinematic viscosity and phase stability when exposed to extreme ambient conditions.

To ensure its suitability before deployment, the anti-emulsification property of the lubricant was evaluated using the ASTM D1401 demulsibility test, along with a visual observation of oil–water separation. The oil exhibited clear phase separation within 10 min, confirming its strong resistance to emulsification. These test results were consistent with the manufacturer’s specifications and ensured stable lubrication during high-pressure operation.

Considering the “cold outside–hot inside” temperature gradient and potential micro-leakage through the seal, No. 10 aviation hydraulic oil was selected as the baseline lubricant. To avoid qualitative descriptions, verifiable quantitative indicators were specified, taking into account actual operating conditions and a reasonable safety margin:

- Low-temperature adaptability: Pour point ≤- 40 °C. In the cold start zone of 0–5 °C, κ = ν/ν₁ ≥ 1 is required to avoid boundary lubrication. Here, ν₁ is calculated based on the bearing’s mean diameter dₘ and rotational speed n, with a baseline estimation of ν₁ ≈ 17 mm²/s.
- High-temperature stability: No significant oxidation or thermal degradation should occur under 200–250 °C, as evaluated by ASTM D943, D2272, or equivalent methods. For this system, the peak oil temperature was ≤ 120 °C, and viscosity retention during cyclic operation was ≥ 90%, with no varnish or deposits observed.
- Viscosity stability: The viscosity index (VI) should be ≥ 120. Within the recommended working temperature window of 0–80 °C, the kinematic viscosity variation should remain within ± 10%, thereby maintaining κ fluctuations within ± 10% to suppress oil film instability caused by temperature changes.
- Anti-emulsification and compatibility: The oil should exhibit short separation times and a minimal emulsion layer volume, as specified in ASTM D1401. It must also be compatible with MoS₂-based anti-wear additives.

**Fig. 1:**
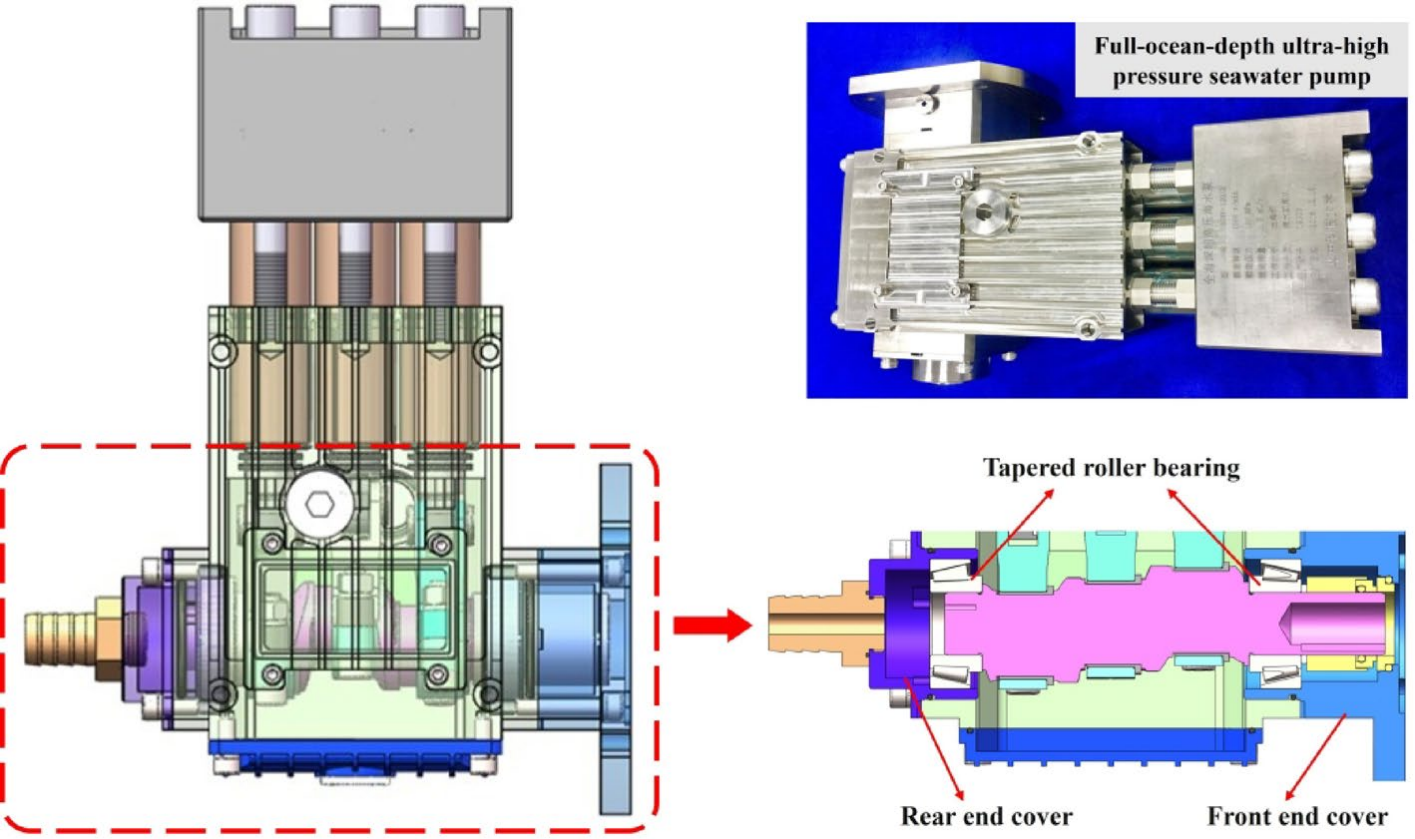
Structural layout of the seawater pump and installation position of rolling bearings.

The bearing installation position in the ultra-high-pressure seawater pump is shown in Fig. 1.

### Experimental procedures and failure inspection

**Fig. 2:**
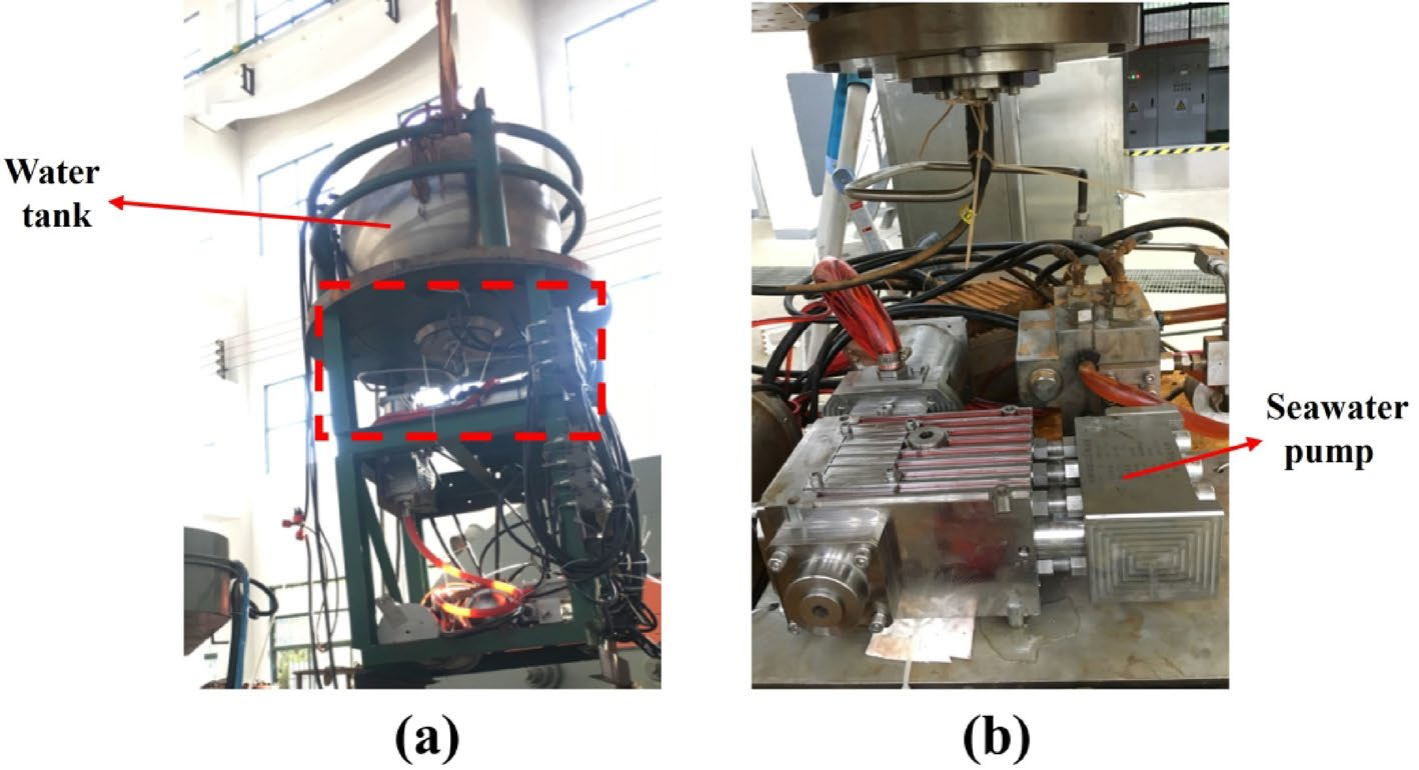
Experimental setup.

To verify the operational stability and structural reliability of the seawater pump system under extreme high-pressure conditions, a high-pressure injection and discharge test was conducted inside a metallic pressure vessel rated at 120 MPa. During testing, the pump was equipped with symmetrical rolling bearings on both sides of the shaft to ensure precise alignment and reduce radial runout. A constant radial load of 2.5 kN was applied to the test bearing through a hydraulic loading mechanism, while axial displacement was constrained by end plates to simulate realistic thrust boundaries. These mechanical boundary conditions were designed to reflect the typical load and constraint conditions of the pump during full-ocean-depth operation. A failure event observed during operation prompted systematic disassembly and inspection of damaged components. The experimental setup is shown in Fig. 2.

## Test procedures and operational failure manifestations

The test was carried out in a cyclic “injection–discharge” mode. The seawater pump system operated under an environmental pressure of 120 MPa. During the first cycle, the system completed one hour of water injection followed by one hour of discharge, and remained stable throughout. In the second cycle, the system ran smoothly during the one-hour discharge phase. However, approximately 50 min into the injection phase, a sudden interruption of the pump motor current was detected, accompanied by a simultaneous pressure drop. Even after depressurization, the motor could not be restarted, indicating a probable mechanical blockage or seizure in the transmission assembly.

It should be noted that the same seawater pump had previously undergone over 20 h of stable operation under 120 MPa on a land-based platform. In total, it had accumulated over 30 h of injection–discharge testing under high-pressure conditions, providing a substantial basis for service validation.

### Disassembly and observation of typical failed components

After the failure event, the seawater pump system was depressurized and disassembled for fault diagnosis. The inner and outer rings of the bearing were extracted for surface inspection. Visual observation and microscopy revealed localized wear bands, spalling zones, and signs of fatigue cracking on the rolling contact surfaces. Additionally, residual lubricant samples from the bearing chamber were found to contain water-in-oil emulsions, indicating a loss of phase stability.

A correlation between operating time and failure progression was observed. In three repeated tests, the transition from normal operation to significant emulsification and friction-induced failure occurred consistently after approximately 30 to 40 h of cumulative run time under 120 MPa. This temporal pattern supports the hypothesis that the degradation of lubrication performance and accumulation of contact fatigue are time-dependent phenomena. The findings highlight the necessity for improved lubricant formulations and more robust material fatigue resistance in long-duration ultra-high-pressure operations.

After system depressurization, the prototype was disassembled for internal inspection. The seawater pump was first disconnected from the motor and valve assembly. Subsequently, the front and rear end caps, pump shaft, sliding sleeve, and rolling bearing assemblies were removed in sequence. The main observations are summarized as follows:

- Shaft seizure and abnormal noise: After external force was applied using tooling, the pump shaft could only rotate slightly. A distinct metallic scraping sound was detected, indicating potential damage to rolling elements or accumulation of metallic debris inside the pump body.
- Bearing fracture and cage deformation: The rear rolling bearing showed extensive fracture of the balls. The cage was severely deformed and torn, with some rolling elements completely disintegrated and displaced.
- PEEK bush embedding and wear: The inner surface of the front PEEK bushing was embedded with numerous metal particles. A typical three-dimensional embedded abrasive wear morphology was observed, along with visible scratches and crushing pits in the contact area.
- Metallic debris accumulation in housing: A large amount of granular and flaky metallic debris was found deposited at the bottom of the housing. Some fragments were traceable to the fractured bearing cage and balls.
- Internal liquid accumulation: A small amount of high-pressure permeated water was retained in the rear cavity of the housing, suggesting that minor leakage during operation was not effectively discharged, which may have triggered internal corrosion or lubricant dilution.

Based on the above findings, the failure was preliminarily attributed to the structural disintegration of the rear rolling bearing, which resulted in the complete seizure of the rotor system. The fragmented bearing balls were likely driven into adjacent components under high pressure, leading to secondary abrasion and cascading damage across multiple parts, severely compromising the system’s recoverability.

### Identification of major failure phenomena

During long-term operation, the bearings of the seawater pump are affected by various factors, which may lead to failure. Bearing failure not only reduces the operating efficiency of the seawater pump but also may cause more severe equipment malfunctions. In this section, several standard failure modes of seawater pump bearings are analyzed. The focus is placed on the mechanisms of lubricant emulsification, surface wear, fatigue cracks, impact damage, and external contamination.

## Lubricating oil emulsification

**Fig. 3:**
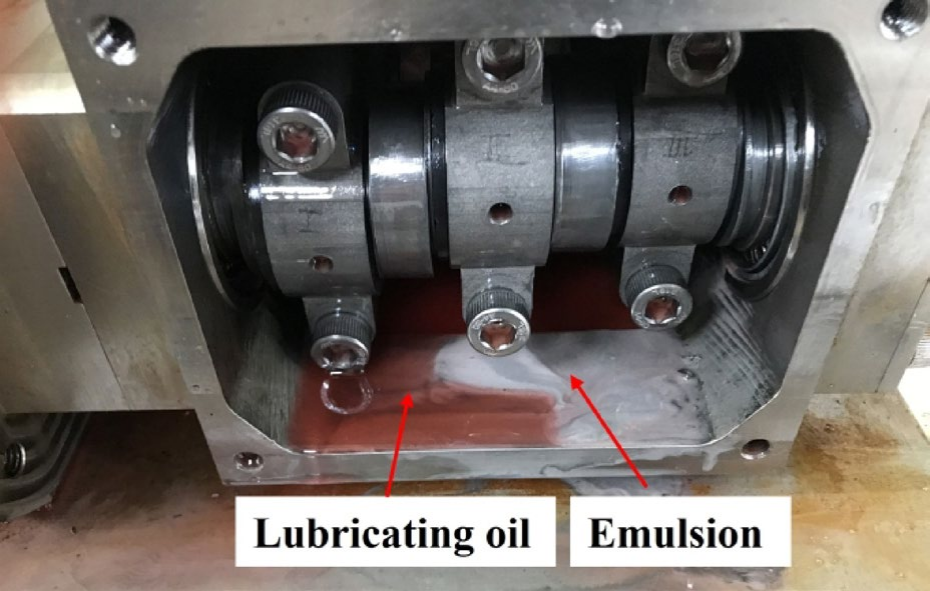
Oil–water emulsification in the lubrication chamber of the seawater pump.

Lubricant emulsification is a critical phenomenon in the failure process of seawater pump bearings. As shown in Fig. 3, significant emulsification of the lubricant was observed in the lubrication chamber after prolonged operation. This phenomenon is caused by the mixing of lubricant and water, typically resulting from leakage at the dynamic seal between oil and seawater. Once water enters the lubricant, continuous agitation by the crank and connecting rod leads to the formation of an emulsion. The emulsion exhibits poor lubricating properties. Its viscosity decreases significantly, and the resulting oil film cannot meet the lubrication requirements of the bearing.

After emulsification, the viscosity of the lubricant is significantly reduced. The oil film can no longer maintain proper sliding between bearing surfaces, which increases friction and wear rate. Under increasing load, the lubricating performance of the emulsion further deteriorates. Sintering or excessive wear may occur on the bearing surface. If the emulsion is used continuously, its corrosiveness may increase. These corrosive substances can accelerate surface damage and may react chemically with bearing materials, leading to the formation of oxide films or corrosion products, further impairing normal bearing function.

Experimental data indicate that the degree of emulsification is closely related to the operating conditions of the seawater pump. The phenomenon is more severe in high-temperature environments or when the seawater has a higher salinity. Therefore, the lubricant replacement interval should be adjusted according to the working environment to avoid long-term effects of emulsification on bearing performance.

### Surface wear and fatigue cracks on bearings

**Fig. 4:**
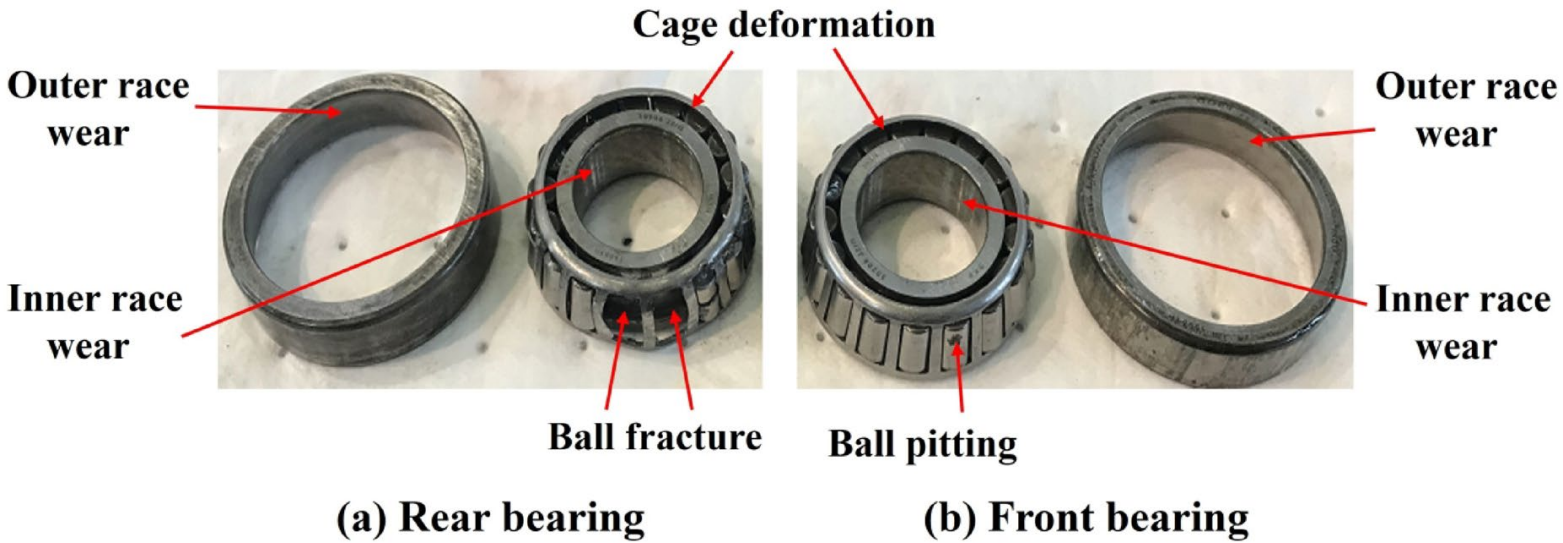
Damage to the inner and outer rings of the bearing.

Surface wear is one of the most common failure modes of seawater pump bearings. As shown in Fig. 4, various degrees of damage were observed during the bearing disassembly process. Severe damage occurred, particularly in the rear bearing. Approximately one-third of the rolling elements in the rear bearing were fractured. The cage was deformed, and visible cracks appeared on some ball surfaces. Localized pitting and noticeable wear marks were found on the front bearing.

Due to prolonged operation under heavy load, the bearing surfaces were subjected to significant wear, with typical wear patterns including surface layer spalling, scratches, and localized indentations. In the early stage of operation, wear remained mild due to adequate lubrication. However, as the lubricant performance deteriorated over time, friction increased, and wear became more severe.

Fatigue cracks in the bearing generally develop under sustained cyclic loads. The formation of fatigue cracks often coincides with surface wear progression. Under repeated loading, microcracks are initiated on the bearing surface. As these cracks propagate, the load-bearing capacity of the bearing is gradually weakened, which may ultimately result in complete failure.

The propagation of fatigue cracks typically follows classical fatigue theory and is characterized by the stress intensity factor, *K*_*IC*_ Material properties, load magnitude, and operating temperature influence the crack growth rate. To extend the fatigue life of bearings, the bearing material and manufacturing process must be carefully optimized. Surface defects should be minimized to reduce the initiation and growth of cracks.

### Impact damage of rolling elements

During the start-up and shutdown phases of seawater pumps, the bearings are often subjected to sudden impact loads. Transient mechanical disturbances during the initiation or cessation of pump operation typically cause these impact loads. Such abrupt load changes may induce localized damage to the bearings. In low-temperature environments, the extent of damage becomes more pronounced. Under impact conditions, the rolling elements and cage are prone to plastic deformation or fracture. As shown in Fig. 4, such damage may lead to complete bearing failure.

Impact-induced damage usually appears most prominently at the contact interfaces, especially between the rolling elements and the cage. When a sudden impact is applied to the bearing, the localized stress can rise sharply, exceeding the material’s yield strength and causing plastic deformation in the affected areas. If not corrected promptly, this damage may result in premature bearing failure.

In theory, impact damage can be simulated using dynamic models. These models consider the bearing’s dynamic stiffness, load distribution, and external impact force. In practice, the occurrence of impact-related damage can be effectively reduced by optimizing bearing design and selecting appropriate materials. This is particularly important in seawater pump systems that undergo frequent start–stop cycles.

### Metal particle ingress and external contamination

Upon disassembly, several metallic particles were observed within the bearing chamber, adhered to raceway surfaces and rolling elements. These contaminants are indicative of particle-induced abrasive wear and localized surface fatigue.

Potential pathways for metallic debris ingress were identified as follows:

(1) Internal frictional wear between piston-cylinder assemblies under high-pressure reciprocation, which generates fine metallic shavings.

(2) Valve seat erosion due to repeated impact and cavitation at the high-pressure inlet and outlet ports.

(3) Seal lip abrasion, particularly at dynamic interfaces, which gradually releases elastomer or metallic particles into the oil circulation.

Due to the absence of inline filtration in the lubrication loop, these particles were able to circulate freely and accumulate in the bearing housing. The presence of hard debris in the lubricant increases the likelihood of surface scratching, three-body abrasion, and pitting initiation under cyclic contact stress. This accelerates rolling contact fatigue and shortens bearing service life.

The findings underscore the importance of incorporating fine filtration and seal integrity monitoring in future design iterations to mitigate contamination-induced failure.

The failure of seawater pump bearings is also frequently associated with external contamination. The sealing system of the bearing is typically designed to prevent the ingress of moisture, dust, and other contaminants. However, in actual applications, seal failure or improper design often allows external contaminants to enter the bearing system, resulting in lubricant contamination.

**Fig. 5:**
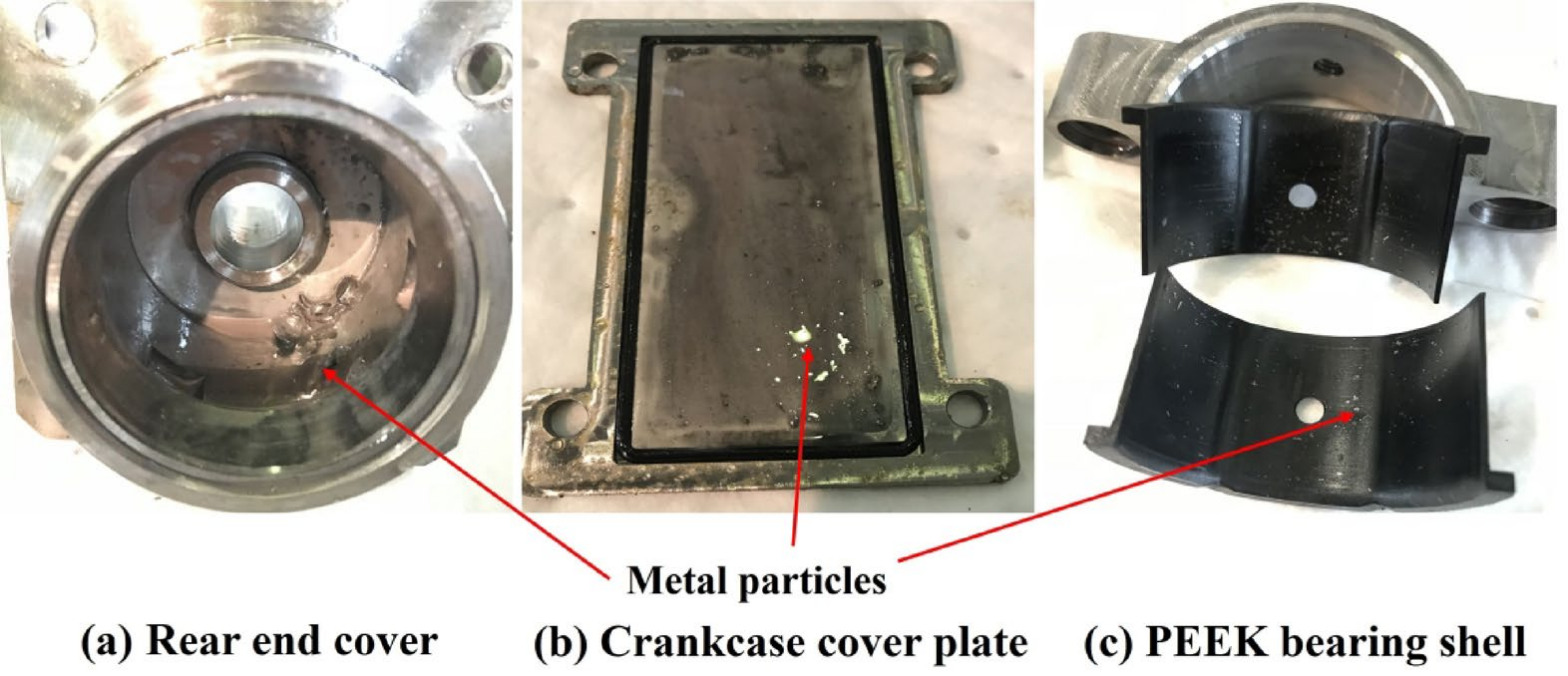
Internal components contaminated with metallic particles.

As shown in Fig. 5, the working environment of the seawater pump contains a large number of metal particles, sand grains, sludge, and other solid debris. Once these particles enter the bearing, they mix with the lubricant and cause abrasive wear. This type of wear significantly damages the bearing surfaces. When hard particles contact the rolling elements, surface indentations, scratches, and material loss are directly induced.

The severity of particulate contamination is closely related to particle size, hardness, and bearing load. Larger or more complex particles can cause more severe surface damage and may even initiate local fatigue cracks. To minimize the impact of particle contamination, the bearing sealing structure must be optimized to prevent the intrusion of external debris. In addition, regular lubricant replacement and system cleaning are essential measures to mitigate abrasive contamination.

### Failure mechanism analysis and root cause determination

The failure of seawater pump bearings is not only influenced by external factors but also closely related to material properties, working environment, and load conditions. A thorough analysis of bearing failure mechanisms can help identify root causes and provide theoretical support for design optimization, material improvement, and service life extension. This section presents a detailed discussion on possible failure mechanisms of seawater pump bearings, with emphasis on lubrication failure, stress concentration, and thermal effects. Both theoretical analysis and numerical simulations are used to construct a comprehensive framework for practical application.

## Lubrication degradation and formation of boundary lubrication regime

Inadequate lubrication is a primary cause of early failure in ultra-high-pressure deep-sea pump bearings. Under high-pressure and heavy-load conditions, the oil film thickness decreases, and the lubrication regime may shift from hydrodynamic lubrication to boundary lubrication, significantly intensifying wear between contact surfaces. In long-duration high-pressure tests conducted on land, it was observed that the lubricant temperature gradually increased with running time, which led to a decrease in viscosity and deterioration of the lubricating performance. Wear occurred on the rolling elements, resulting in increased clearance between balls and raceways. Consequently, vibrations and impacts were introduced, eventually triggering bearing failure.

The lubrication state of a bearing is typically evaluated by the viscosity ratio κ, which is defined as follows^40^:

2 $$\kappa =\frac{\nu }{{{\nu _1}}}$$

Where κ is the lubrication condition indicator (viscosity ratio); ν is the actual kinematic viscosity of the lubricant at operating temperature, in mm²/s; and ν_1_ is the rated viscosity, which is a function of bearing diameter and rotational speed, also in mm²/s.

**Fig. 6:**
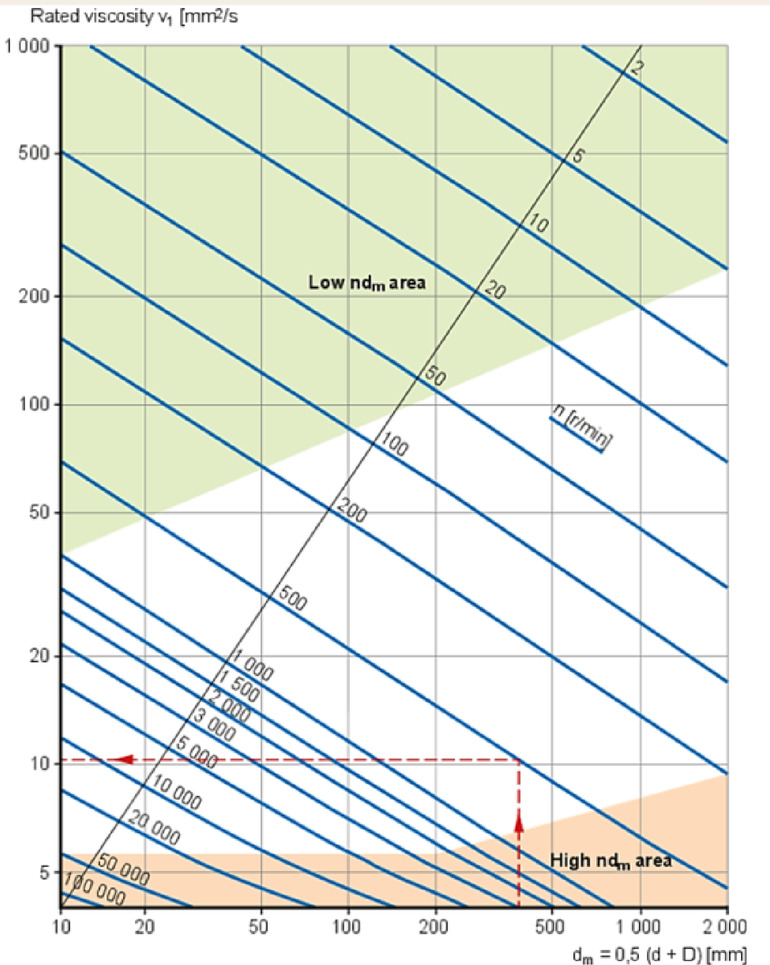
Chart for calculating the rated viscosity.

Figure 6 illustrates the method for determining *ν*_1_ based on bearing dimensions and speed. In general, lubrication is considered sufficient when κ > 1, whereas κ < 1 indicates possible oil film insufficiency and the risk of boundary lubrication or even dry friction. Most engineering guidelines recommend operating bearings within the range of κ = 1 to 4 to ensure service life and reliability. In this study, the selected bearing has an outer diameter of 62 mm and an inner diameter of 30 mm, operating at 1500 rpm. Aviation hydraulic oil No. 10, with a viscosity of approximately 10 mm²/s at 40 °C, was used as the lubricant. According to Fig. 6, the rated viscosity *ν*_1_ required under this condition is approximately 17 mm²/s. Therefore, the calculated viscosity ratio is:

3 $$\kappa =\frac{{10}}{{17}} \approx 0.59$$

Clearly, the calculated value is lower than the recommended minimum of 1, indicating insufficient lubrication and a significant risk of bearing failure. This issue is more pronounced in deep-sea environments, where seawater temperatures typically range from 2 to 4 °C. The low ambient temperature further reduces the initial oil temperature, increasing viscosity lag and flow resistance during startup. As a result, boundary friction is more likely to occur during cold start conditions. Two possible failure modes are proposed as follows:

### Oil film rupture

Oil Film Rupture.

The proper operation of bearings relies on the presence of a continuous lubricant film, which effectively separates contact surfaces and reduces direct contact and wear. During operation, the oil film may break due to the combined effects of load, speed, and temperature. Once rupture occurs, the friction at the contact surface increases rapidly, resulting in localized heating and wear.

The formation of the lubricating oil film can be described using the Reynolds Eq. 4^1^:

4 $$h=\frac{{3\mu u}}{P}$$

Where *h* is the oil film thickness, *µ* is the dynamic viscosity of the lubricant, *u* is the relative speed of the contact surfaces, and *P* is the applied load pressure. When the film thickness falls below a critical threshold, direct metal-to-metal contact occurs, leading to premature wear and potential failure.

### Oil degradation and emulsification

The quality and lubricating performance of oil are strongly influenced by its physical properties. Seawater pump bearings often operate in harsh and humid environments. Under such conditions, seawater contamination may occur, leading to oil–water emulsification. Emulsification reduces the oil’s viscosity and alters its tribological behavior, thereby preventing the formation of a stable oil film. As a result, adequate lubrication cannot be maintained, which significantly accelerates bearing wear.

The degree of oil emulsification can be analyzed using the logarithmic mixing rule^42^:

5 $$\log {\eta _{emuls}}={\phi _o}\log {\eta _o}+{\phi _w}\log {\eta _w}$$

Where *η*_*emuls*_ is the dynamic viscosity of the emulsion; *η*_*o*_ and *η*_*w*_ are the viscosities of oil and water, respectively; *ϕ*_*o*_ and *ϕ*_*w*_ are the volume fractions of oil and water (satisfying *ϕ*_*o*_ + *ϕ*_*w*_ = 1). The viscosity of the emulsion is generally lower and cannot effectively resist friction between bearing surfaces, thus accelerating wear.

#### Stress Concentration–Induced local material failure

Under deep-sea high-pressure conditions, stress concentration becomes a critical factor in bearing failure. The repeated cyclic loads and complex contact geometry cause localized stress amplification at the roller–raceway interface.

To evaluate the effect of external pressure, a series of finite element simulations were performed. The results indicate that as ambient pressure increased from 40 MPa to 120 MPa, the compressive preload on the outer ring was significantly enhanced. This shift intensified the contact pressure between rolling elements and the raceway surfaces.

Notably, when external pressure exceeded 100 MPa, the equivalent von Mises stress at the bearing shoulder region increased by approximately 15%, compared to the baseline at 40 MPa. This stress amplification was most prominent near the contact zone of the inner race, where micro-pitting and fatigue cracks were later observed in failure analysis.

The altered stress distribution under high-pressure exposure not only accelerated subsurface fatigue but also weakened the load-bearing capacity of the lubricant film, promoting surface damage initiation.

During operation, bearings are subjected to both external loads and internal mechanical forces. Under high load conditions, the internal stress distribution within the bearing often becomes non-uniform. Due to geometrical irregularities or material defects, stress concentration may occur in certain regions. These localized stress zones are prone to initiate crack formation and propagation, eventually leading to material failure.

(1) Fatigue Failure.

Fatigue failure is one of the most common failure modes in bearing materials. When bearings are subjected to cyclic loading over an extended period, microcracks gradually accumulate on the material’s surface. These cracks expand with time and eventually cause a fracture. The growth rate of fatigue cracks can be described using the Paris law^43^:

6 $$da/dN=C{\left( {\Delta K} \right)^m}$$

Where *da*/*dN* is the crack growth rate, Δ*K* is the stress intensity factor range, and *C* and m are material-dependent constants. This equation can be used to predict the crack propagation rate under different stress conditions. For seawater pump bearings, frequent load fluctuations significantly aggravate fatigue damage, especially under high-load and high-speed operating conditions.

(2) Stress Corrosion Cracking (SCC).

In addition to mechanical loading, the marine environment surrounding seawater pump bearings tends to induce corrosion. Seawater contains a high concentration of chloride ions and other corrosive species, which can initiate electrochemical corrosion on the bearing surface. When combined with tensile stress, corrosion accelerates the initiation and propagation of stress corrosion cracking. The stress level and the concentration of the corrosive medium typically influence the crack growth rate in SCC.

The following model can express the crack growth rate under SCC conditions^43^:

7 $$\frac{{da}}{{dt}}={K_{SCC}}{\left( {{\sigma _{eff}}} \right)^n}$$

Where *da*/*dt* is the crack propagation rate, *K*_*SCC*_ is the stress corrosion constant, *σ*_*eff*_ is the effective stress, and *n* is a constant related to material and environmental factors. This equation can be used to describe the corrosion cracking behavior of seawater pump bearings under different operating conditions.

**Fig. 7:**
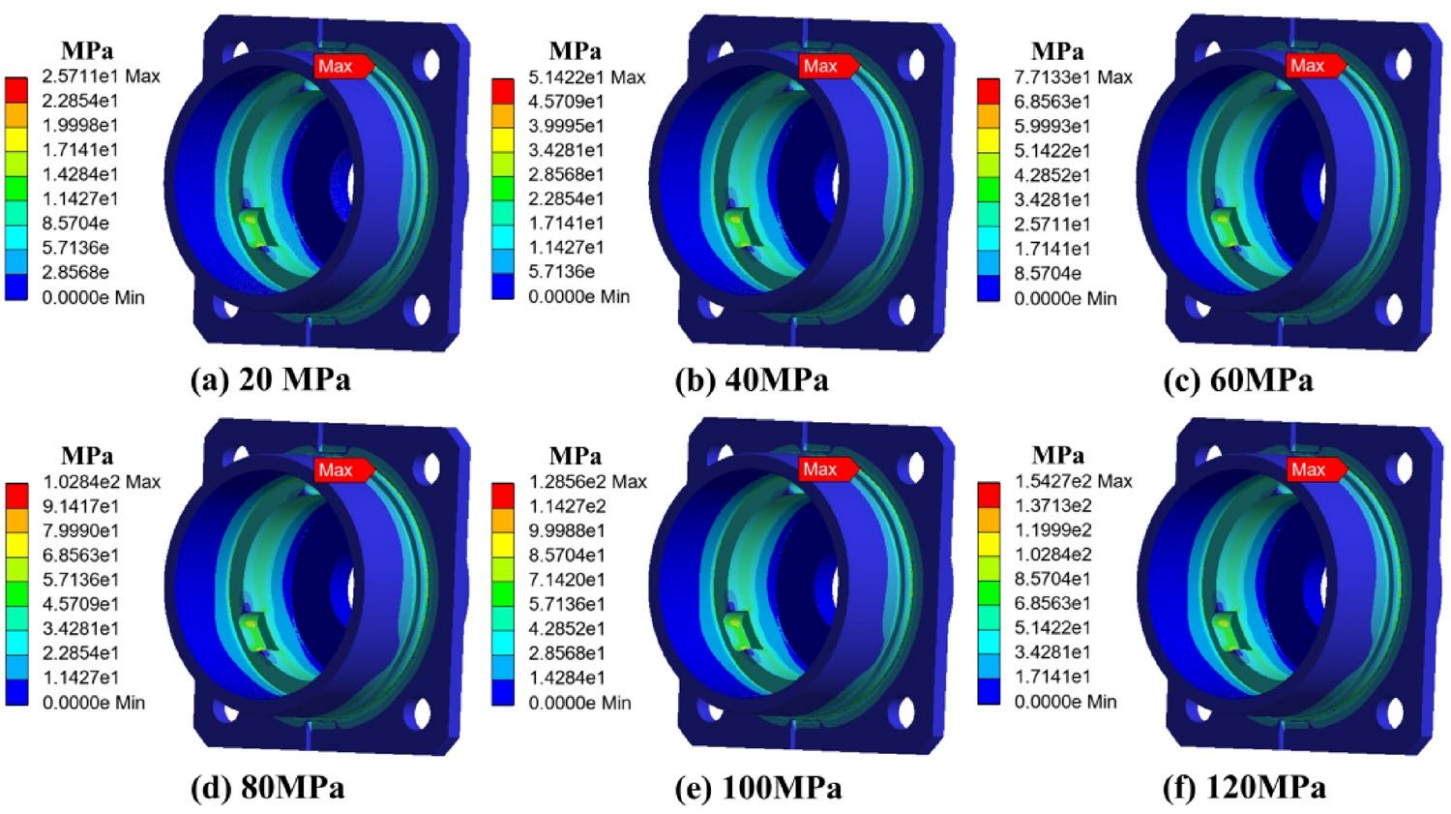
Stress distribution of the end cover under different ambient pressures.

**Fig. 8:**
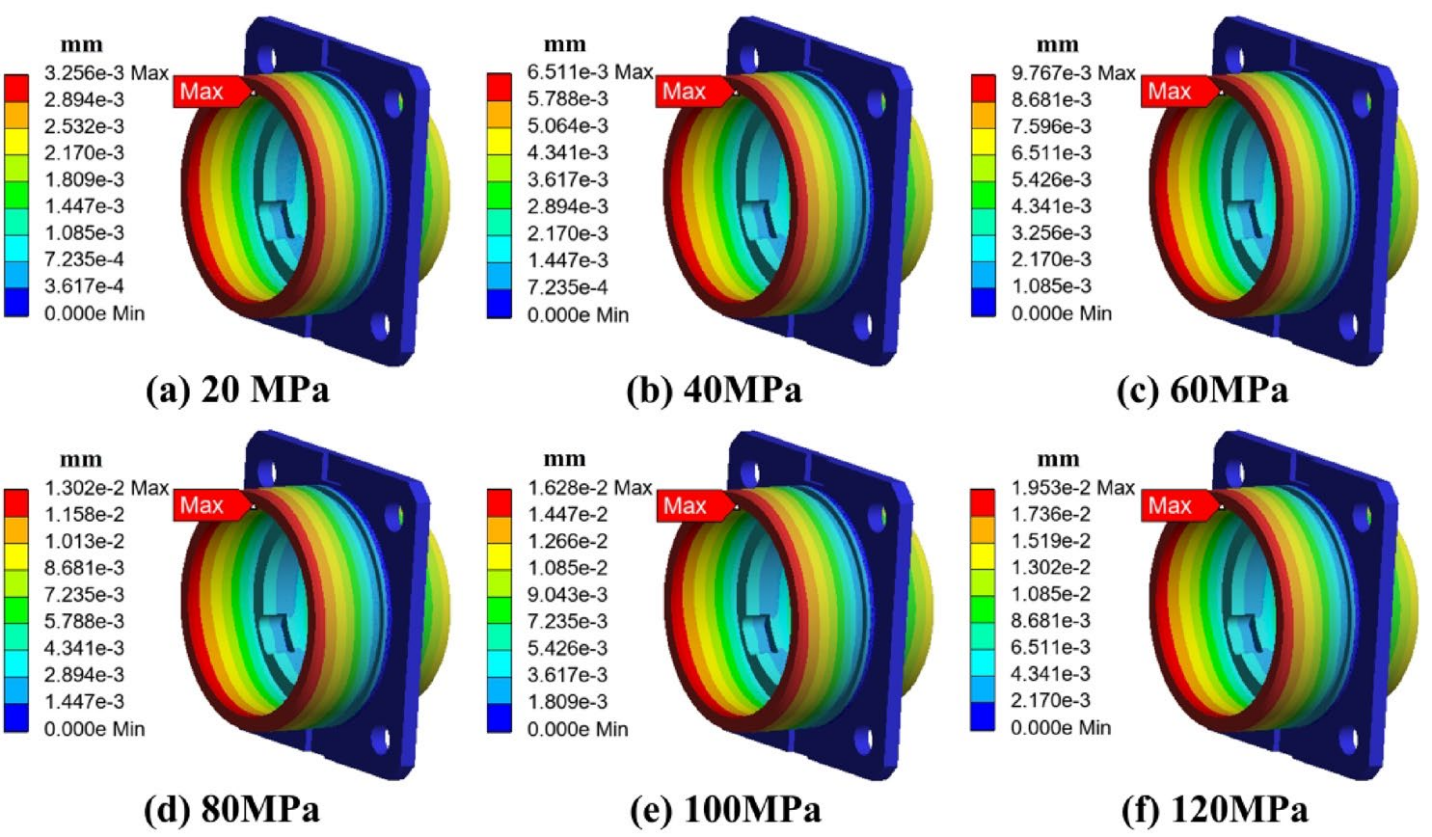
Strain distribution of the end cover under different ambient pressures.

**Fig. 9:**
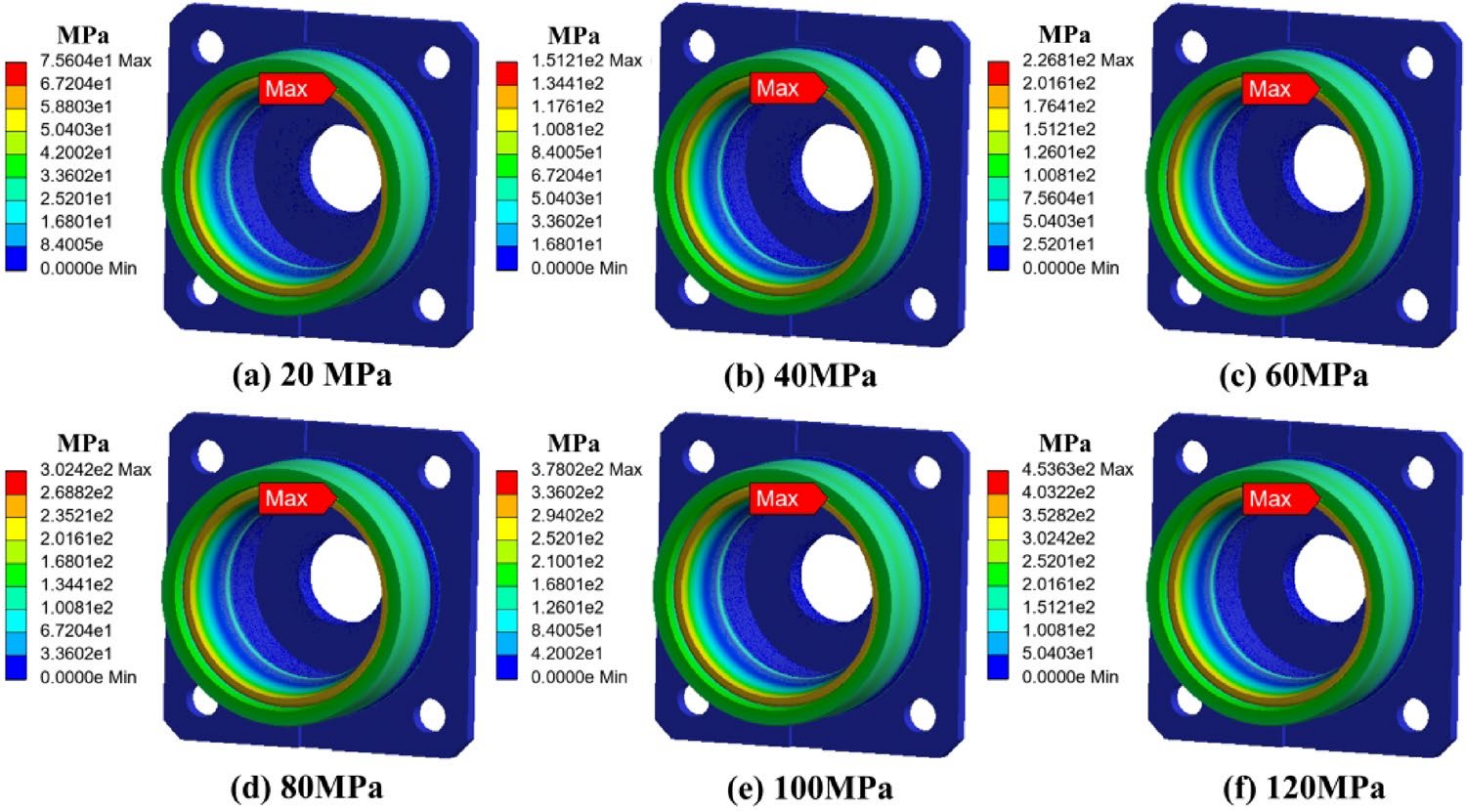
Stress distribution of the bearing and end cover under different ambient pressures.

**Fig. 10:**
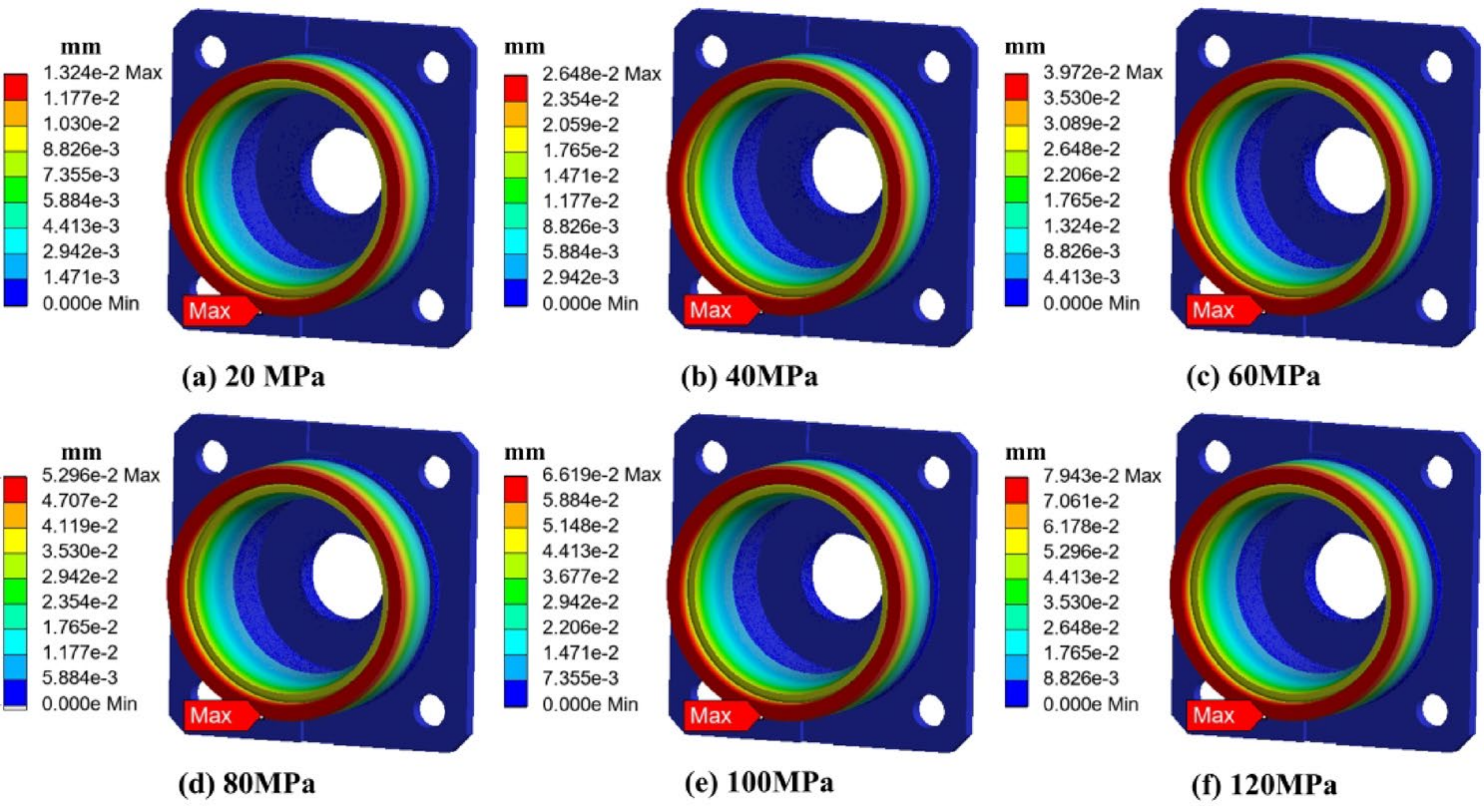
Strain distribution of the bearing and end cover under different ambient pressures.

To systematically evaluate the stress and deformation behavior of the end cap and outer ring structure under high-pressure environments, finite element simulations were conducted. Two configurations were analyzed: the end cap alone and the combined end cap–outer ring structure. The applied external pressure was gradually increased from 0 MPa to 120 MPa. Stress and strain distributions were extracted at each stage. The results are shown in Figs. 7, 8, 9 and 10.

As shown in Fig. 7 (stress distribution of the end cap), under external pressure ranging from 0 to 120 MPa, the overall stress exhibited a trend of concentration from the edge toward the transition zone. With increasing pressure, the stress peak gradually appeared in the stepped transition region where the end cap contacts the outer ring. Due to geometric discontinuity, local stress concentration was observed in this region. However, the maximum stress remained below the material’s yield strength, indicating that the end cap operated entirely within the elastic regime. This result reveals that the end cap design provides sufficient redundancy against uniformly distributed external pressure, and short-term shock or high-pressure conditions are unlikely to trigger yielding failure.

As shown in Fig. 8 (strain distribution of the end cap), the strain was also mainly concentrated in the same transition region, consistent with the stress pattern. The strain value increased approximately linearly with external pressure. The maximum strain was minimal, not exceeding 20 microns, indicating that the deformation was limited and insufficient to cause permanent geometric distortion. The strain contour further revealed minor radial shrinkage at the mating surface between the end cap and the outer ring. However, the shrinkage amplitude was only in the tens of microns, which can be considered negligible in terms of its influence on the assembly clearance.

As shown in Fig. 9 (stress distribution of the combined structure), after the end cap was embedded into the outer ring to form the combined structure, the stress distribution under pressure became more uniform. The outer ring effectively shared part of the external load, reducing the stress peak in the transition region of the end cap by approximately 15%. The local stress concentration effect was significantly alleviated. This indicates that the outer ring not only bore the significant pressure load but also played a structural shielding role, thereby improving the compressive stability of the entire assembly.

As shown in Fig. 10 (strain distribution of the combined structure), the radial deformation of the outer ring increased linearly with pressure. At 120 MPa, the maximum deformation was only several tens of microns, which is less than 0.1% of the 62 mm outer ring diameter. This minor deformation was entirely within the elastic range and far below the threshold that could cause interference or mismatch during assembly. Furthermore, the inner ring and rolling elements would undergo simultaneous micro-compression under the same pressure, ensuring that the geometric fit of the entire system remained stable.

Although stress concentration was indeed observed in the transition zone between the end cap and outer ring, the stress values did not exceed the yield strength, and the strain amplitude was minimal. The simulation results confirmed that the main effect under high pressure was elastic deformation rather than plastic failure. This ensured that no sudden change in mating clearance or rolling element obstruction would occur. Therefore, it can be clearly concluded that bearing early failure due to structural deformation of the end cap or outer ring is effectively ruled out. This conclusion is not only consistent with post-disassembly inspections but also provides a solid basis for subsequent analysis of the failure mechanism.

#### Thermal effects on structural performance of bearings

Under ultra-high-pressure operating conditions, thermal effects play a crucial role in the degradation of bearing performance. Frictional heat generation at the contact surfaces between the rollers and raceways causes localized temperature rise, which in turn affects lubricant properties and material fatigue behavior.

Although direct temperature measurements during the test were not available due to structural constraints, a qualitative estimation was made based on published literature and energy dissipation considerations. Under boundary lubrication induced by oil emulsification, the friction coefficient increases significantly, leading to localized thermal accumulation.

It is estimated that the temperature near the roller–raceway contact zone could reach 70–80 °C. Such elevated temperatures accelerate the oxidative degradation of the base oil and reduce its dynamic viscosity. As a result, the load-bearing capacity of the lubricant film diminishes, increasing metal-to-metal contact risk.

This thermal feedback loop—friction-induced heating, viscosity drop, and enhanced wear—contributes to a cascading failure process. The correlation between temperature rise and mechanical wear emphasizes the necessity of both thermal control and lubricant stability in high-pressure applications.

Thermal effects are a critical factor influencing the performance of seawater pump bearings. During operation, friction generates heat, leading to a localized rise in temperature. This phenomenon becomes more pronounced under high-load and high-speed conditions. The temperature increase can degrade the lubricant’s performance, thereby accelerating bearing wear. Simultaneously, the temperature rise can induce thermal expansion and structural changes in the material, further affecting the load-bearing capacity of the bearing.

(1) Thermal Expansion Effect.

Bearings are typically made of metallic materials, which generally possess a high coefficient of thermal expansion. When the temperature increases, the dimensions of the bearing change. Under heavy loads, such thermal expansion may alter the internal clearance of the bearing, thereby affecting its operational accuracy. If the contact clearance between bearing components becomes either too small or too large, the lubricant film may rupture or become uneven, resulting in increased wear.

(2) Thermal Fatigue.

Thermal fatigue occurs due to stress generated by rapid temperature fluctuations, which can initiate and propagate cracks within the material. During the start-up and shutdown phases of the pump, severe temperature variations may trigger thermal fatigue. The crack propagation associated with thermal fatigue follows classical fatigue crack growth models and tends to be more severe at elevated temperatures.

Thermal effects played a critical role in the failure progression of the bearing system. During high-pressure operation, frictional heating at the rolling contact interfaces caused a gradual temperature rise in the bearing chamber. This temperature increase had a direct impact on lubricant stability and material behavior.

A coupled degradation mechanism was identified: as the lubricant operated under marginal conditions, friction-induced heat led to elevated oil temperatures. This thermal input accelerated the emulsification process and caused a reduction in kinematic viscosity. The deteriorated lubricant film was no longer able to maintain full separation of metal surfaces, resulting in increased direct contact, elevated shear stress, and intensified surface fatigue.

Simultaneously, the thermal expansion of bearing components introduced internal dimensional changes, reducing operational clearances and aggravating stress concentrations at contact points. This closed-loop interaction between temperature rise, mechanical loading, and lubricant degradation formed a feedback cycle, which significantly contributed to the onset of wear, micro-cracking, and eventual seizure. This integrated analysis clarifies the thermomechanical pathway toward catastrophic bearing failure under ultra-high-pressure deep-sea conditions.

#### Integrated analysis and attribution of dominant mechanisms

Based on a comprehensive analysis of typical failure morphologies, lubrication state evaluation, and high-pressure finite element simulation results, it can be clearly concluded that the boundary lubrication condition caused by lubricant degradation is the fundamental mechanism leading to early bearing failure, rather than local stress concentration in the end cover–outer ring structure.

(1) Lubrication system degradation is the primary cause.

The observed phenomena, including raceway spalling, roller scratches, and cage wear, were all closely associated with oil film failure. The measured viscosity of the lubricant was significantly lower than the minimum service requirement. Combined with common emulsification effects in seawater environments, it is inferred that the bearing operated under boundary lubrication or even partial dry friction conditions for extended periods. Under such conditions, the friction coefficient between metallic contact surfaces increases sharply, triggering a series of coupled damage processes including surface fatigue, localized wear, and heat accumulation.

(2) Thermo-mechanical coupling accelerates damage evolution.

In the boundary lubrication regime, frictional heating at metal contact interfaces leads to a continuous rise in surface temperature. This localized heating drives the material into a high-strain state governed by thermo-mechanical coupling, reducing its yield strength. Even if the overall load remains within rated design limits, such conditions may still initiate low-cycle fatigue cracks under high-frequency cyclic stress. These cracks typically originate from the raceway or roller surfaces and propagate inward, eventually resulting in spalling or fracture.

(3) Structural stress concentration is not the dominant failure mechanism.

Although finite element simulations revealed some degree of stress concentration at the stepped transition between the end cover and the outer ring, the peak stress remained well below the material’s yield strength, and the associated strain amplitudes were limited to several tens of microns. These levels were insufficient to cause permanent deformation or misalignment at mating surfaces. Furthermore, the structural enveloping effect of the outer ring helped to mitigate stress concentration further, enabling an overall elastic response. These findings are consistent with the absence of deformation or misfit observed during disassembly inspection. Therefore, it can be confirmed that structural stress concentration is not the leading cause of early failure, and the related design possesses sufficient structural redundancy.

(4) Contaminant particles and impact loads as amplifying factors.

Under poor sealing conditions in the lubrication system, fine particles in seawater may enter the lubrication chamber and cause abrasive wear on metal contact surfaces. In addition, pressure fluctuations and impact loads during start-up and shutdown may accelerate the evolution of incipient damage into macroscopic failure zones. Although these external factors are not the root cause, they exhibit a typical synergistic amplification effect.

## Targeted improvement measures and performance enhancement

### Lubrication system optimization: incorporation of molybdenum disulfide additive

To improve the lubrication performance under ultra-high-pressure conditions, a MoS₂-based anti-wear additive was introduced into No. 10 aviation hydraulic oil. The modified lubricant demonstrated enhanced mechanical efficiency and surface protection during testing.

All comparative tests between the base oil and the additive-enhanced oil were conducted under identical conditions to ensure result reliability. The bearing type (33206 heavy-duty tapered roller bearing), oil volume (150 mL), system pressure (120 MPa), and operating duration (28 h) were held constant across all trials. The only variable was the presence or absence of the MoS₂ additive.

This strict control of experimental parameters ensures that any observed differences in bearing wear, emulsification suppression, or mechanical efficiency can be attributed directly to the additive effect. Such a design enhances the interpretability and reproducibility of the performance improvement analysis.

Molybdenum disulfide (MoS₂), as a high-performance anti-wear additive, is widely used in lubricating oils to enhance lubrication performance. Its unique lubricating property originates from its molecular structure. Within the MoS₂ crystal, sulfur atoms and molybdenum atoms form slip layers. These layers can create a compelling lubricating film between frictional surfaces, thereby reducing direct metal-to-metal contact and minimizing both friction and wear. The anti-wear mechanisms of MoS₂ additives can be summarized as follows::

(1) Reduction of the friction coefficient:

A solid lubricating film formed by MoS₂ on friction surfaces decreases the real contact area between metal components. As a result, the friction coefficient is reduced, which leads to lower wear rates of bearings and related parts.

(2) Enhanced load-carrying capacity:

The molecular structure of MoS₂ provides self-lubricating properties. Even under extreme pressure, its slip layers remain stable, thereby improving the load-bearing capacity of the lubricant and preventing oil film rupture.

(3) Lower temperature rise:

Due to the reduced friction, the temperature rise in bearings and other lubricated components can be effectively controlled. This prevents oil degradation and surface damage caused by excessive thermal stress.

These advantages allow MoS₂ additives to significantly improve the lubrication condition of seawater pump bearings, reduce wear, and enhance mechanical efficiency. The performance is especially remarkable under high-load and high-pressure situations.

**Fig. 11:**
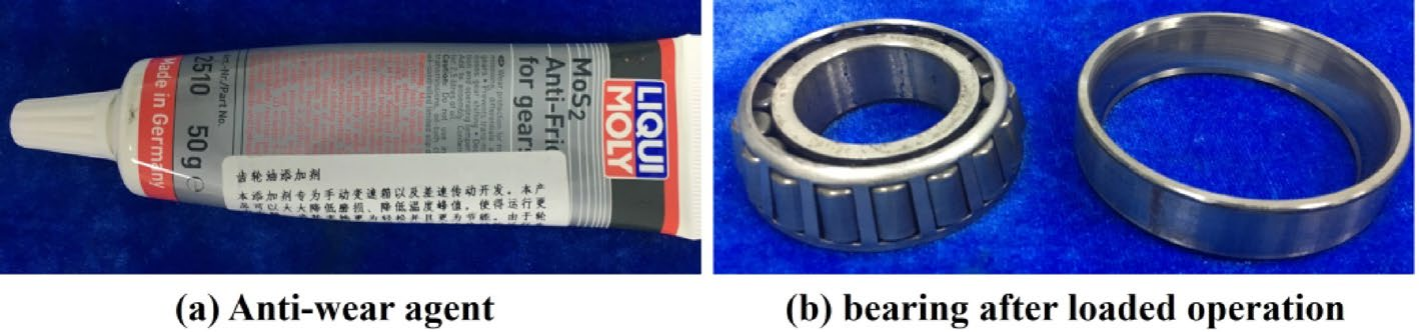
Bearings operating under load after the addition of anti-wear additives.

**Fig. 12:**
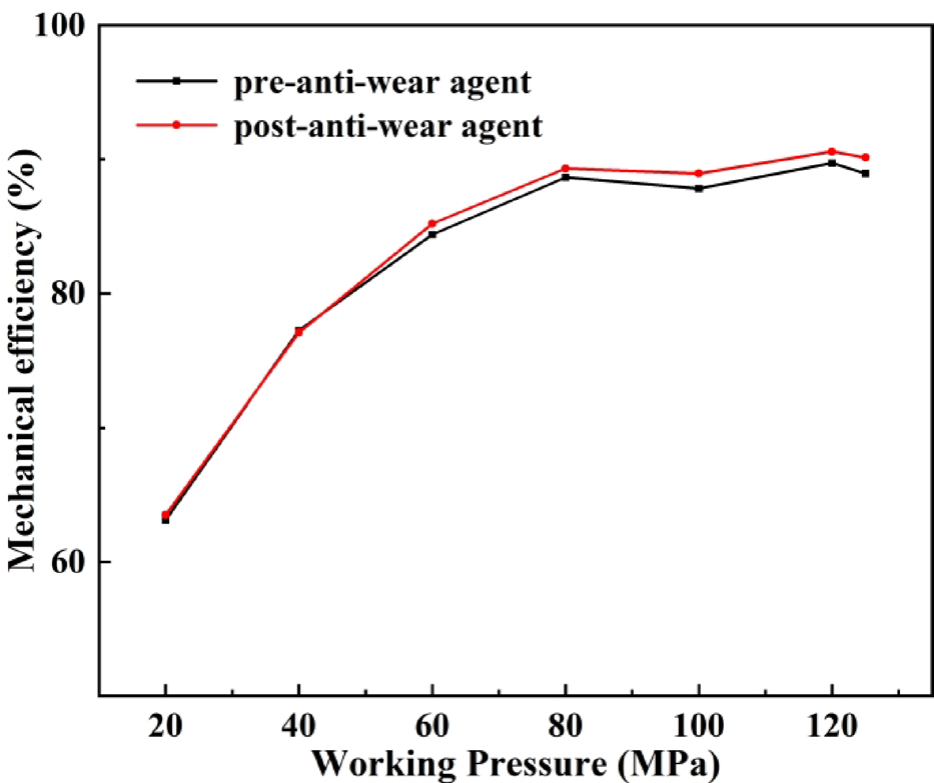
Mechanical efficiency of the seawater pump before and after adding anti-wear additives.

In this study, MoS₂ anti-wear additive from LIQUI MOLY (Germany) was added to No. 10 aviation hydraulic oil. A series of performance tests was conducted under different output pressures. The experimental results showed a clear improvement in the lubrication performance after the addition of MoS₂. As shown in Fig. 11, wear marks on bearing contact surfaces were significantly suppressed. Bearing wear was also reduced, resulting in improved overall mechanical efficiency of the seawater pump. Figure 12 presents the comparative mechanical efficiency under various output pressures, with and without the MoS₂ additive.

As shown in Fig. 12, with increasing output pressure, the mechanical efficiency of the seawater pump was gradually improved after the addition of MoS₂ anti-wear additive. The improvement was particularly evident under ultra-high working pressure. This indicates that MoS₂ can effectively enhance lubrication conditions, reduce friction and wear, and thereby improve the mechanical efficiency and reliability of the seawater pump.

After the addition of MoS₂, the performance of the lubricating oil was significantly enhanced, as reflected in the following aspects:

(1) Enhanced lubrication performance:

The MoS₂ additive effectively improved the load-carrying capacity of the lubricating oil. Under high-pressure conditions, a protective film formed by MoS₂ on friction surfaces prevented oil film rupture and reduced emulsification. The presence of the lubricating layer minimized direct metal-to-metal contact, leading to a significant reduction in friction and wear. As a result, the service life of the bearing was extended.

(2) Reduced friction coefficient:

MoS₂ formed a thin solid film on friction interfaces, which decreased the coefficient of friction. According to the experimental data, the improvement in mechanical efficiency directly reflected this reduction. Lower friction not only increased transmission efficiency but also reduced heat generation. This helped lower the operating temperature of the bearings and prevented lubricant oxidation and degradation caused by excessive heat.

(3) Controlled temperature rise:

As friction and wear decreased, the temperature rise in bearings and other mechanical components was significantly reduced. Experimental results showed that the overheating of the crankcase was alleviated after MoS₂ was added. This further confirmed that the lubricant performance had been improved, enhancing the operational stability of the seawater pump.

Although the addition of MoS₂ significantly improved lubrication performance, some limitations still exist. Under certain extreme conditions, the viscosity of the lubricant or the synergistic effect of other additives may not reach the optimal level. Therefore, future work can focus on the following directions for further improvement:

(1) Lubricant formulation optimization:

In addition to MoS₂, other anti-wear and extreme-pressure additives, such as lithium-based or molybdenum-based compounds, can be explored to enhance overall lubricant performance further.

(2) Multi-stage lubrication system design:

A multi-stage lubrication system could be developed according to the operating conditions of the seawater pump. This system could adapt lubricant type and delivery based on load and temperature variations, ensuring sufficient lubrication under all conditions.

(3) Optimization of oil change intervals:

The replacement cycle of the lubricant can be optimized by considering additive life and lubricant degradation trends. This would help prevent equipment failure due to the performance decline of the lubricant.

### Bearing structure upgrade: replacement with Heavy-Duty rolling bearings

The rated speed of the seawater pump is 1500 r/min. The maximum radial load of a single plunger is 6 kN, while the maximum radial load capacity of a single bearing is 4.5 kN. Although the originally designed tapered roller bearing 30,206 meets the preliminary requirements, its basic dynamic load rating and basic static load rating are relatively low, at 50 kN and 44 kN respectively. These ratings cannot guarantee normal operation of the seawater pump under long-term high-pressure and high-load conditions. Furthermore, due to the insufficient viscosity of the lubricating oil, the oil film thickness in the bearing is inadequate, which further aggravates bearing wear and fatigue.

To address this issue, the original bearing was replaced with a heavy-duty tapered roller bearing 33,206. This replacement aims to improve the bearing’s load capacity and lubrication conditions, thereby enhancing the operational stability and service life of the seawater pump. The selection of the heavy-duty rolling bearing was based on a detailed analysis of the seawater pump’s operating conditions, particularly the bearing’s load capacity requirements under high load and prolonged operation. Compared with the original model 30,206, the heavy-duty tapered roller bearing 33,206 exhibits significant improvements in load capacity, specifically:

(1) The basic dynamic load rating was increased from 50 kN to 79.7 kN;

(2) The basic static load rating was increased from 44 kN to 76.5 kN;

(3) The fatigue load limit was increased from 4.8 kN to 8.5 kN;

(4) The limiting speed remains 11,000 r/min.

The bearing’s load capacity directly affects the seawater pump’s performance, especially under prolonged high-pressure output conditions. After the replacement with the heavy-duty tapered roller bearing, the contact length between the rollers and the bearing inner and outer rings was increased from 9.33 mm to 16.2 mm. This indicates a significant increase in the bearing contact area, which helps reduce the pv value of the bearing friction pair (i.e., the product of contact pressure and sliding speed), thereby reducing friction and wear and enhancing the bearing’s load capacity.

Under constant oil viscosity, the increase in roller length directly leads to an increase in the minimum oil film thickness. The increased oil film thickness optimizes the lubrication conditions between the bearings, effectively reducing friction between the bearing and raceway, decreasing heat generation, and preventing excessive consumption and degradation of the lubricating oil. According to lubrication theory, the oil film thickness can be estimated by the following formula^44^:

8 $${h_{\hbox{min} }}=\frac{L}{{\sqrt {2 \cdot \pi \cdot \eta \cdot P} }}$$

Where *h*_min_ is the minimum oil film thickness, *L* is the effective length of the roller, is the viscosity of the lubricating oil, and *P* is the contact pressure of the bearing.

As the effective length of the roller increases, the oil film thickness also increases. This improves the lubrication effect and further enhances the operational stability and service life of the bearing.

After replacing with the heavy-duty tapered roller bearing, the contact area between the rollers and the raceway is significantly increased. This change directly affects the distribution of the bearing’s contact stress and maximum von Mises stress. Due to the increased contact area, the maximum contact stress between the rollers and the raceway is reduced. This further decreases the risk of fatigue damage. The reduction in contact stress directly improves the bearing’s fatigue life. According to classical contact mechanics theory, when the contact stress decreases, the rate of material fatigue damage is reduced, thus extending the bearing’s fatigue life. This change can be further verified by the distribution of von Mises stress^45^:

9 $${\sigma _{eq}}=\sqrt {\sigma _{1}^{2}+\sigma _{2}^{2} - {\sigma _1}{\sigma _2}+3\tau _{{xy}}^{2}}$$

Where *σ*_*eq*_ is the equivalent stress, *σ*_1_ and *σ*_2_ are the principal stresses, and *τ*_*xy*_ is the shear stress.

**Fig. 13:**
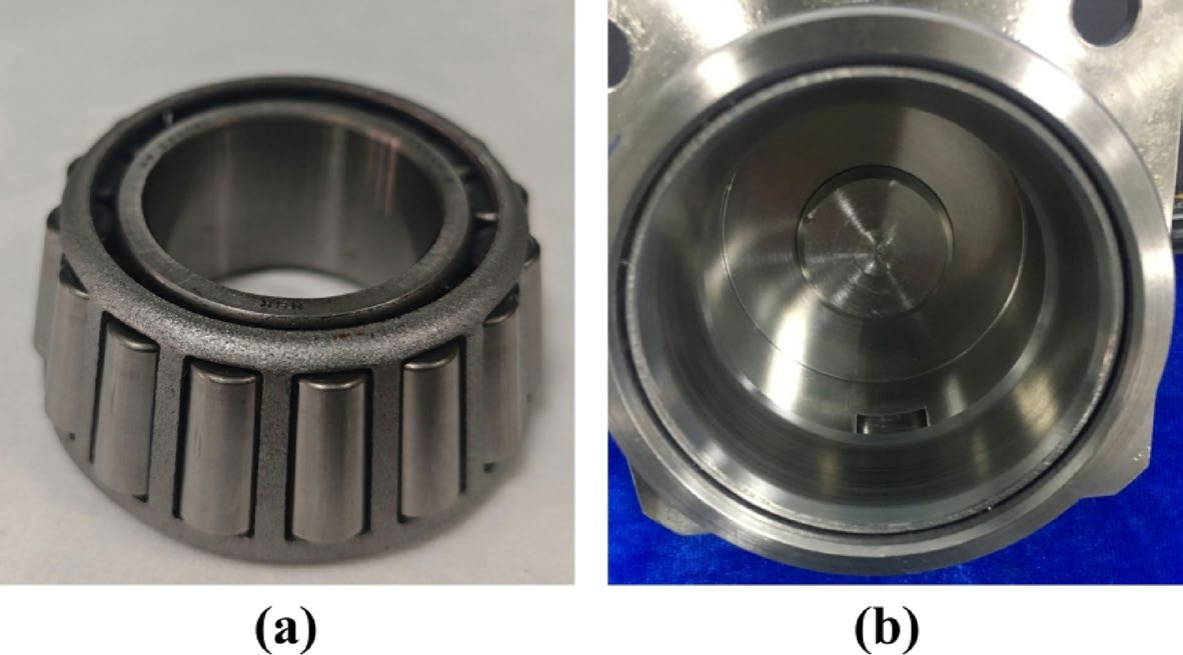
Inner and outer rings of the bearing after replacement with heavy-duty bearings.

By calculating the von Mises stress for different bearing designs, it can be found that the increase in roller length effectively improves the bearing’s fatigue life. In actual operation, after the seawater pump was equipped with the heavy-duty tapered roller bearing 33,206, a 28-hour running test was conducted under a working pressure of 120 MPa. As shown in Fig. 13, the wear marks on the bearing outer ring surface were significantly reduced. Compared to the condition before replacement, the wear was markedly improved. This result verifies the advantages of the heavy-duty bearing in terms of load capacity and lubrication performance.

## Conclusion

This study focused on the sudden failure of rolling bearings during the operation of a full-ocean-depth ultra-high-pressure seawater pump under an extreme working pressure of 120 MPa. The failure mechanisms were systematically analyzed, and two targeted improvement measures were proposed to enhance the system’s operational reliability and service life.

First, to address the insufficient load capacity of the original 30,206 tapered roller bearing, a larger 33,206 heavy-duty bearing was adopted. This replacement offered significant advantages in both rated load and fatigue life, thereby improving pressure-bearing capacity and operational stability. Second, to reduce wear risks caused by lubrication degradation, the lubrication system was optimized by adding molybdenum disulfide (MoS₂) anti-wear additive to No. 10 aviation hydraulic oil. This effectively reduced frictional losses and enhanced mechanical efficiency. Experimental verification showed that the improved system operated continuously for 28 h without failure under 120 MPa, with stable mechanical efficiency and reduced surface wear. These results confirmed the effectiveness of the proposed solutions.

The findings provide both theoretical support and engineering guidance for bearing selection and lubrication system design in high-pressure deep-sea seawater pumps. Future research can further focus on optimizing bearing materials and structures, developing intelligent lubrication control strategies, and establishing condition monitoring methods under extreme conditions, to support the long-term and reliable operation of deep-sea equipment.

## Data Availability

The data that support the findings of this study are available from the corresponding author upon reasonable request.

## References

[1] Tian, Y., Zhang, C. & Yang, L. Analysis of the longitudinal coupled dynamic characteristics of shaft-shell system considering the lubrication of thrust bearing. *Eur. J. Mech. A. Solids*. **113**, 105726. 10.1016/j.euromechsol.2025.105726 (2025).

[2] Kamarapu, S. K., Amarnath, M. & Tiwari, S. Nanoenhanced biolubricant for improving lubrication in roller bearing steel-steel contact surfaces - a comparative tribological study. *Diam. Relat. Mater.* **154**, 112160. 10.1016/j.diamond.2025.112160 (2025).

[3] Wu, Y. et al. Vibration properties of full ceramic bearing under elastohydrodynamic fluid lubrication based on the energy approach. *Case Stud. Therm. Eng.* **64**, 105459. 10.1016/j.csite.2024.105459 (2024).

[4] Mo, S. et al. Research on thermal-lubrication-mechanics coupling dynamic characteristic for rolling bearing. *Commun. Nonlinear Sci. Numer. Simul.* **148**, 108931. 10.1016/j.cnsns.2025.108931 (2025).

[5] Lu, G. et al. Micro-morphological analysis, lubricating behaviors, and wear failure characteristics and mechanisms of propeller hub bearings in marine environments. *Wear* **530–531**, 205047. 10.1016/j.wear.2023.205047 (2023).

[6] Zhang, Y. et al. Nonlinear dynamic response and stability of piezoelectric shells with piezoelectric nonlinearities. *Int. J. Mech. Sci.* **304**, 110731. 10.1016/j.ijmecsci.2025.110731 (2025).

[7] Li, X. et al. Dynamic modeling of a spline-shaft system including time-varying fretting friction. *Int. J. Mech. Sci.* **294**, 110192. 10.1016/j.ijmecsci.2025.110192 (2025).

[8] Yang, R., Zhang, Z. & Chen, Y. Analysis of vibration signals for a ball bearing-rotor system with raceway local defects and rotor eccentricity. *Mech. Mach. Theory*. **169**, 104594. 10.1016/j.mechmachtheory.2021.104594 (2022).

[9] Li, X. et al. Dynamic modeling and vibration analysis of double row cylindrical roller bearings with irregular-shaped defects. *Nonlinear Dyn.* **112**, 2501–2521. 10.1007/s11071-023-09164-5 (2024).

[10] Yang, Z. et al. Lubrication state monitoring of sliding bearing based on triboelectric Stribeck curve. *Nano Energy*. **140**, 111059. 10.1016/j.nanoen.2025.111059 (2025).

[11] Niu, P. et al. Study on Splash oil supply and lubrication characteristics of Conrod small end bearing with C-shaped oil collection structure. *Tribol. Int.* **210**, 110768. 10.1016/j.triboint.2025.110768 (2025).

[12] Zhang, C., Pan, L., Wang, S., Wang, X. & Tomovic, M. An accelerated life test model for solid lubricated bearings used in space based on time-varying dependence analysis of different failure modes. *Acta Astronaut.* **152**, 352–359. 10.1016/j.actaastro.2018.08.027 (2018).

[13] Chang, Z., Jia, Q., Yuan, X. & Chen, Y. Main failure mode of oil-air lubricated rolling bearing installed in high speed machining. *Tribol. Int.* **112**, 68–74. 10.1016/j.triboint.2017.03.024 (2017).

[14] Wei, S. et al. Tribological performance of journal bearings for fuel oil lubrication based on Ni-coated graphite hybrid microparticle and carbon fiber reinforced polytetrafluoroethylene composites. *J. Mater. Res. Technol.* **36**, 7410–7421. 10.1016/j.jmrt.2025.05.024 (2025).

[15] Hu, J. et al. The influence of the connecting rod bearing failure on the bearing temperature of marine low-speed engines. *Eng. Fail. Anal.* **109772**10.1016/j.engfailanal.2025.109772 (2025).

[16] Hong, J., Liu, F., Ma, Y., Chen, X. & Wang, Y. Composite failure analysis of an aero-engine inter-shaft bearing inner ring. *Eng. Fail. Anal.* **165**, 108707. 10.1016/j.engfailanal.2024.108707 (2024).

[17] Tian, J., Wang, P., Xu, H., Ma, H. & Zhao, X. Nonlinear vibration characteristics of rolling bearing considering flexible cage fracture. *Int. J. Non-Linear Mech.* **156**, 104478. 10.1016/j.ijnonlinmec.2023.104478 (2023).

[18] Salunkhe, V. G. & Desavale, R. G. An intelligent prediction for detecting bearing vibration characteristics using a machine learning model. *J. Nondestructive Evaluation Diagnostics Prognostics Eng. Syst.* **4**10.1115/1.4049938 (2021).

[19] Salunkhe, V. G., Khot, S. M., Yelve, N. P., Jagadeesha, T. & Desavale, R. G. Rolling element bearing fault diagnosis by the implementation of Elman neural networks with long Short-Term memory strategy. *J. Tribol.* **147**10.1115/1.4067382 (2025).

[20] Salunkhe, V. G., Khot, S. M., Desavale, R. G., Yelve, N. P. & Jadhav, P. S. An integrated dimension theory and modulation signal bispectrum technique for analyzing bearing fault in industrial fibrizer. *J. Nondestructive Evaluation Diagnostics Prognostics Eng. Syst.* **7**10.1115/1.4065545 (2024).

[21] Salunkhe, V. G., Khot, S. M., Desavale, R. G. & Yelve, N. P. Unbalance bearing fault identification using highly accurate Hilbert–Huang transform approach. *J. Nondestructive Evaluation Diagnostics Prognostics Eng. Syst.* **6**10.1115/1.4062929 (2023).

[22] Jagadeesha, T. et al. in *Advances in Industrial Automation and Smart Manufacturing.* (eds A. Arockiarajan, M. Duraiselvam, & Ramesh Raju) 631–645 (Springer Singapore).

[23] Salunkhe, V. G., Desavale, R. G., Khot, S. M. & Yelve, N. P. A novel incipient fault detection technique for roller bearing using deep independent component analysis and variational modal decomposition. *J. Tribol.* **145**10.1115/1.4056899 (2023).

[24] Kong, W. & Li, H. Remaining useful life prediction of rolling bearing under limited data based on adaptive time-series feature window and multi-step ahead strategy. *Appl. Soft Comput.* **129**, 109630. 10.1016/j.asoc.2022.109630 (2022).

[25] Wang, Y., Zhao, J., Yang, C., Xu, D. & Ge, J. Remaining useful life prediction of rolling bearings based on pearson correlation-KPCA multi-feature fusion. *Measurement* **201**, 111572. 10.1016/j.measurement.2022.111572 (2022).

[26] Dai, L., Guo, J., Wan, J. L., Wang, J. & Zan, X. A reliability evaluation model of rolling bearings based on WKN-BiGRU and wiener process. *Reliab. Eng. Syst. Saf.* **225**, 108646. 10.1016/j.ress.2022.108646 (2022).

[27] Schmidt, S., Heyns, P. S. & Gryllias, K. C. A discrepancy analysis methodology for rolling element bearing diagnostics under variable speed conditions. *Mech. Syst. Signal Process.* **116**, 40–61. 10.1016/j.ymssp.2018.06.026 (2019).

[28] Shinde, P. V. & Desavale, R. G. Application of dimension analysis and soft competitive tool to predict compound faults present in rotor-bearing systems. *Measurement* **193**, 110984. 10.1016/j.measurement.2022.110984 (2022).

[29] Vishwendra, M. A. et al. A novel method to classify rolling element bearing faults using K-Nearest neighbor machine learning algorithm. *ASCE-ASME J. Risk Uncert Engrg Sys Part. B Mech. Engrg*. **8**10.1115/1.4053760 (2022).

[30] Yang, Z., Hong, J., Wang, D., Ma, Y. & Cheng, R. Failure analysis of an aero-engine inter-shaft bearing due to clearance between the outer ring and its housing. *Eng. Fail. Anal.* **150**, 107298. 10.1016/j.engfailanal.2023.107298 (2023).

[31] Shinde, P. V., Desavale, R. G., Jadhav, P. M. & Sawant, S. H. A multi fault classification in a rotor-bearing system using machine learning approach. *J. Brazilian Soc. Mech. Sci. Eng.* **45**, 121. 10.1007/s40430-023-04015-1 (2023).

[32] Shi, Z., Zhang, G., Yan, C., Li, X. & Liu, J. Dynamic modeling of cylindrical roller bearings considering raceway crack defects. *Mech. Syst. Signal Process.* **237**, 112981. 10.1016/j.ymssp.2025.112981 (2025).

[33] Shinde, P. V., Desavale, R. G., Deshmukh, S. S., Patil, S. M. & Patil, V. R. Dynamic behavior and numerical investigation of contact characteristics of rotor bearing system using dimensional analysis and finite element model. *J. Fail. Anal. Prev.* **25**, 1600–1613. 10.1007/s11668-025-02191-x (2025).

[34] Palit, P., Sri, P., Gokarn, P., Kumar, A. & Das, S. Rolling contact fatigue failure analysis of ball bearing in gear box. *J. Fail. Anal. Prev.* **25**, 62–69. 10.1007/s11668-025-02109-7 (2025).

[35] Raj, K. K., Kumar, S. & Kumar, R. R. Systematic review of bearing component failure: strategies for diagnosis and prognosis in rotating machinery. *Arab. J. Sci. Eng.* **50**, 5353–5375. 10.1007/s13369-024-09866-x (2025).

[36] Mali, A. R. et al. A novel method for bearing fault diagnosis based on novel feature sets with machine learning technique. *J. Tribol.* **147**10.1115/1.4066306 (2024).

[37] Patil, S. S. et al. Intelligent fault diagnosis based on the EAO-VMD in Dual-Rotor cylindrical roller bearings. *J. Tribol.* **148**10.1115/1.4068831 (2025).

[38] Li, R. et al. Failure analysis of a needle roller bearing in a megawatt reciprocating pump. *J. Fail. Anal. Prev.* **24**, 108–115. 10.1007/s11668-023-01821-6 (2024).

[39] Luan, X. et al. Rolling bearing fault diagnosis method based on mean singular value screening. *J. Mech. Sci. Technol.* **39**, 13–26. 10.1007/s12206-024-1202-x (2025).

[40] Jakobsen, M. O. et al. Vibration signatures in ball bearings as a function of lubricant viscosity ratio κ, under alternating lubrication conditions. *Tribol. Int.* **156**, 106840. 10.1016/j.triboint.2020.106840 (2021).

[41] Lombera Rodríguez, H. & Tello, J. I. in *In Mathematics Applied To Engineering, Modelling, and Social Issues*. 1–43 (eds Smith, F. T.) (Springer International Publishing, 2019). John N. Mordeson.

[42] Sousa, A. M., Pereira, M. J. & Matos, H. A. Oil-in-water and water-in-oil emulsions formation and demulsification. *J. Petrol. Sci. Eng.* **210**, 110041. 10.1016/j.petrol.2021.110041 (2022).

[43] Pugno, N., Ciavarella, M., Cornetti, P. & Carpinteri, A. A generalized paris’ law for fatigue crack growth. *J. Mech. Phys. Solids*. **54**, 1333–1349. 10.1016/j.jmps.2006.01.007 (2006).

[44] Dou, P. et al. Review of ultrasonic-based technology for oil film thickness measurement in lubrication. *Tribol. Int.* **165**, 107290. 10.1016/j.triboint.2021.107290 (2022).

[45] Chen, J. et al. Statistical correlation neural network for small sample data and its engineering application: predicting maximum mises stress on ultrasonic-assisted grinding surfaces. *Measurement* **253**, 117823. 10.1016/j.measurement.2025.117823 (2025).

